# m6A Methylation Regulates Osteoblastic Differentiation and Bone Remodeling

**DOI:** 10.3389/fcell.2021.783322

**Published:** 2021-12-21

**Authors:** Mei Huang, Shaozhe Xu, Lifei Liu, Miao Zhang, Jianmin Guo, Yu Yuan, Jiake Xu, Xi Chen, Jun Zou

**Affiliations:** ^1^ School of Kinesiology, Shanghai University of Sport, Shanghai, China; ^2^ School of Sports Science, Wenzhou Medical University, Wenzhou, China; ^3^ School of Physical Education and Sports Science, South China Normal University, Guangzhou, China; ^4^ School of Biomedical Sciences, University of Western Australia, Perth, WA, Australia

**Keywords:** m6A methylation, bone remodeling, osteoporosis, bone marrow mesenchymal stem cells, signaling pathways

## Abstract

Osteoporosis is a prevalent bone disease of the aging population, which is characterized by a decrease in bone mass because of the imbalance of bone metabolism. Although the prevention and treatment of osteoporosis have been explored by different researchers, the mechanisms underlying osteoporosis are not clear exactly. N6 methyladenosine (m6A) is a methylated adenosine nucleotide, which functions through its interaction with the proteins called “writers,” “readers” and “erasers.” The epigenetic regulation of m6A has been demonstrated to affect mRNA processing, nuclear export, translation, and splicing. At the cellular level, m6A modification has been known to affect cell proliferation, differentiation, and apoptosis of bone-related cells, such as bone marrow mesenchymal stem cells (BMSC), osteoblasts, and osteoclasts by regulating the expression of ALP, Runx2, Osterix, VEGF, and other related genes. Furthermore, PTH/Pth1r, PI3K‐Akt, Wnt/β‐Catenin, and other signaling pathways, which play important roles in the regulation of bone homeostasis, are also regulated by m6A. Thus, m6A modification may provide a new approach for osteoporosis treatment. The key roles of m6A modification in the regulation of bone health and osteoporosis are reviewed here in this article.

## 1 Introduction

Epigenetic modifications regulate gene expression and translation and affect cell development and differentiation (Kohli and Zhang, 2013). Epigenetic abnormalities can occur in different ways, including DNA, RNA, and histone modification (Litt et al., 2001; Akhavan-Niaki and Samadani, 2013; Xu et al., 2016; Roignant and Soller, 2017). RNA transmits DNA genetic information to proteins and participates in biological processes via RNA post-transcriptional modification. Previous studies have identified more than 150 types of RNA modifications (Helm and Motorin, 2017). Among them, N6-methyladenosine (m6A) modification is the most common gene modification in mammalian cells, occurring in the adenosine base at the nitrogen-6 position of mRNAs (Desrosiers et al., 1974; Wei et al., 1975). The core sequence of m6A is RRm^6^ACH ([G/A/U] [G > A]m^6^ AC [U > A > C]), which is located in the 3′ untranslated region (3′UTR) adjacent to the stop codon of mRNA (Dominissini et al., 2012; Meyer et al., 2012). Unlike other gene modifications, the modification of m6A is dynamically reversible and regulates the maturation, translation, and degradation of precursor mRNAs (Haussmann et al., 2016; Guo et al., 2017; Yu et al., 2018). m6A RNA methylation participates in the development of diseases, as an increase in m6A promotes the expression of oncoproteins. Studies have revealed that the high prevalence of m6A can enhance the proliferation, invasion, and survival of cancer cells, including cancer cells of gastric, lung, breast, and liver (Zhang et al., 2016; Du et al., 2017; Cai et al., 2018; Chen et al., 2019; Lin et al., 2019).

Recent studies have shown that m6A methylation is involved in the development of bone-related diseases such as osteoporosis (Wu et al., 2018), osteoarthritis (Liu et al., 2019), and osteosarcoma (Miao et al., 2019; Wang et al., 2019). Osteoporosis is a bone metabolic disease with a reduction in bone mass and degradation of bone structure, which increases the risk of bone fracture (Felsenberg and Boonen, 2005). With the growth of the aging population worldwide, the prevalence of osteoporosis is increasing rapidly, and the number of patients is estimated to be more than 200 million at present (Tian et al., 2017; Shapiro et al., 2019). The patient’s bones gradually become fragile and can easily fracture, which seriously affects people’s life span and quality of life (Muftic et al., 2013; Shapiro et al., 2019). m6A RNA methylation plays a crucial role in regulating bone formation and resorption by influencing cytokines, hormones, and signaling pathways. This study reviews the influence of m6A on osteoporosis, particularly its relationship with bone homeostasis through multiple mechanisms.

## 2 Basic Introduction of m6A Methylation

m6A is one of the most prevalent internal modifications in eukaryotic messenger RNA. m6A regulates gene expression through affecting the translocation, exporting, translation, and decay of RNA (Huang et al., 2020). Thus, dynamic m6A modification is important for many physiological processes. The abundance and function of m6A are effected by the interaction of methyltransferases (“writers”), binding proteins (“readers”), and demethylases (“erasers”) (Panneerdoss et al., 2018; Shi et al., 2019).

### 2.1 Writers

Writers transfer a methyl group to the N-6 position of adenosine. N-methyladenosine (mA) is mainly catalyzed by the m6A methyltransferase complex, which encompasses Wilms tumor 1-associated protein (WTAP), methyltransferase-like 3 (METTL3), and methyltransferase-like14 (METTL14) (Ping et al., 2014). METTL3 plays a major catalytic role in regulating alternative splicing of mRNAs (Ke et al., 2017; Xu et al., 2017; Feng et al., 2018), while METTL14 assists in RNA substrate binding (Wang et al., 2016). WTAP is required for the METTL3-MELLT14 complex to be located in nuclear speckles and catalyzes the activation of m6A methyltransferase *in vivo* (Ping et al., 2014).

Recently, an increasing number of other components of the methyltransferase complex has been found, such as KIAA1429 (VIRMA, vir-like m6A methyltransferase associated) (Schwartz et al., 2014), methyltransferase-like protein 16 (METTL16) (Warda et al., 2017), RNA binding motif protein 15 (RBM15), RBM15B (Patil et al., 2016), and zinc finger CCCH-type containing 13 (ZC3H13) (Wen et al., 2018). These proteins interact with the methyltransferase complex to regulate the stability of the complex and affect m6A methylation of mRNAs (Knuckles et al., 2018). However, comprehension of m6A methyltransferase is still exploratory, so it remains further research on these writers.

### 2.2 Readers

Readers modulate the stability and translation of m6A-modified RNAs (Wang et al., 2014; Wang et al., 2015). The most common type of m6A “reader” proteins is the YTH family, including YTHDF1, YTHDF2, YTHDF3, YTHDC1, and YTHDC2, which contain the unique YTH domain and directly bind to m6A to regulate downstream targets (Luo and Tong, 2014; Xu et al., 2014; Kasowitz et al., 2018). Among them, YTHDF3 mainly attenuated methylated mRNAs and then decreased translation through cooperation with YTHDF1 and YTHDF2. Thus, these three YTHDF proteins interact and coordinate to regulate methylated mRNAs (Shi et al., 2017). The second type of “reader” proteins are the heterogeneous nuclear ribonucleoprotein (HNRNP) family proteins (HNRNPA2B1, HNRNPC, HNRNP G), which regulate the maturation of RNA substrates in the nucleus (Alarcón et al., 2015; Liu et al., 2015). With more studies focusing on m6A methylation, other RNA-binding proteins (Readers) have been found, such as insulin-like growth factor 2 mRNA-binding proteins (IGF2BP) (Huang et al., 2018), leucine-rich pentatricopeptide repeat-containing (LRPPRC), and fragile X mental retardation 1 (FMR1) (Zhang et al., 2018). The potential number of readers is large and m6A modifications depend on readers to fulfill biological functions, which contains a broad research space.

### 2.3 Erasers

Demethylase (“erasers”) can remove the methyl group of m6A off RNAs, indicating that the methylation of m6A is a dynamic process and is reversible. There are two common demethylases: fat mass and obesity-associated protein (FTO) and alkB homolog 5 (ALKBH5) (Jia et al., 2011; Zheng et al., 2013). FTO was first reported related to body mass and obesity in humans (Dina et al., 2007; Zhao et al., 2014). In 2011, Jia et al. (2011) found that FTO is partially located on nuclear speckles and that m6A in nuclear RNA is the physiological substrate of FTO. FTO removes m6A methylation in RNAs to affect physiological activities such as glycolysis (Qing et al., 2021) and adipogenesis (Wang et al., 2020a). FTO depletion induces a notable increase in the total m6A levels of polyadenylated RNAs. ALKBH5 also localizes to the nucleus and significantly impacts mRNA export and RNA metabolism through demethylation activity. Alkbh5-deficient male mice showed increased m6A mRNA expression, which impairs fertility through aberrant spermatogenesis and apoptosis (Zheng et al., 2013). At present, few proteins exhibit demethylation activity. The functions and mechanisms of additional m6A demethylases still need further mining.

## 3 Regulation of m6A Methylation on Bone Cells

Human bones undergo remodeling through bone formation and resorption, and the coordination between osteogenesis and osteoclastogenesis maintains bone health (Felsenberg and Boonen, 2005). Any disruption to this balance leads to bone-related diseases, including osteoporosis, which is mainly characterized by bone mass loss, reduction of bone strength, and increased risk of fractures (Bliuc et al., 2015; Onsensus Development, 2001). Several studies have shown that m6A methylation plays an essential role in regulating bone cells, including bone marrow mesenchymal stem cells (BMSCs) and osteoblasts (Wu et al., 2018; Yu et al., 2019a; Mi et al., 2020; Yan et al., 2020). Thus, m6A methylation may open a new approach for the prevention and treatment of osteoporosis.

### 3.1 Regulation of m6A Methylation on Bone Marrow Mesenchymal Stem Cells

BMSCs are multiple differentiation potential cells that can differentiate into osteoblasts, chondrocytes, and bone marrow adipocytes. BMSCs play an essential role in human skeletal health by balancing osteogenic and lipogenic differentiation (Kawai et al., 2009; Chen et al., 2016). The preferential differentiation of mesenchymal stem cells into adipocytes leads to an increase in bone marrow fat and a decrease in osteoblasts and osteocytes, resulting in bone mass loss and even the development of osteoporosis (Rosen et al., 2009; Scheller and Rosen, 2014).

As METTL3 plays a crucial role in catalyzing m6A methylation, previous studies have primarily focused on regulating METTL3-mediated m6A methylation on osteogenesis. Recently, Wu et al. (2018) demonstrated that conditional knockout of METTL3 in BMSCs increased bone loss, leading to impairment of bone formation and development of the pathological characteristics of osteoporosis in mice, indicating that downregulation of METTL3-mediated m6A methyltransferase in BMSCs induced osteoporosis. The findings further revealed that the dysregulation of m6A methyltransferase increased adipocyte differentiation and decreased osteoblast differentiation, resulting in a reduction in osteogenesis. Mechanistically, METTL3-mediated m6A methyltransferase targeted Pth1r (parathyroid hormone receptor-1) and reduced protein translation, impaired the function of PTH (parathyroid hormone)-Pth1r signaling, and dysregulated BMSC-derived osteoblasts (Wu et al., 2018). Tian et al. (2019) also discovered that downregulation of METTL3 decreased the early and later osteoblast differentiation in BMSCs, as both ALP activity and mineralized nodules were reduced, indicating that downregulation of METTL3-mediated m6A methyltransferase affects osteoblast differentiation in BMSCs. Research revealed that as the downstream target of m6A methyltransferase after the knockdown expression of METTL3, the expression of osteogenic-related genes such as Runx2 and Osterix was reduced (Tian et al., 2019). Furthermore, the reduction of Akt phosphorylation and downregulation of the PI3K-Akt signaling pathway also regulate METTL3-mediated m6A on bone formation (Marie, 2012; Tian et al., 2019). Consistently, the knockdown of METTL3 in BMSCs increased adipocyte differentiation. Yao et al. (2019) demonstrated that silencing METTL3 in porcine BMSCs decreased Janus kinase1 (JAK1) mRNA m6A modification levels and promoted adipogenesis through the JAK1/STAT5/C–EBPβ signaling pathway. These results demonstrated that the downregulation of METTL3 in BMSCs suppressed osteoblast differentiation and promoted adipocyte differentiation, leading to decreased bone formation and even the development of osteoporosis.

On the contrary, overexpression of METTL3 increased osteogenic differentiation and remedied BMSC dysfunction in ovariectomized mice by directly promoting the m6A methylation of Runx2 to maintain the stability of mRNA Runx2, leading to a high expression level of Runx2. In addition, m6A methylation of precursor miR-320 indirectly amplified the effect of METTL3 overexpression on osteogenesis through the downregulation of mature miR-320 in BMSCs. Furthermore, downregulation of mature miR-320 levels protected against METTL3 silence-induced bone loss *in vivo* (Yan et al., 2020).

In addition, m6A methylation affects bone formation through blood vessels. Previous studies have found that vascular endothelial growth factor (VEGF), including three homologous spliced variants, 120, 164, and 188, promote angiogenesis and osteogenesis (Breier et al., 1992; Wallner et al., 2015; Hu and Olsen, 2016; Tong et al., 2019). Tian et al. (2019) illustrated that knockdown of METTL3 reduced the expression of VEGFA (VEGFA-164 and VEGFA -188). Previous studies have shown that VEGFA-164 and VEGFA -188 promote the proliferation and differentiation of osteoblasts from BMSCs (Carmeliet et al., 1999), suggesting that METTL3 also regulates bone formation through m6A methylation of VEGF in BMSCs, followed by the mutual promotion of angiogenesis and osteogenesis in bone (Ramasamy et al., 2014).

Further research showed that METTL3 promoted the activation of m6A methylation of MYD88-RNA in menstrual blood-derived mesenchymal stem cells (MenSCs), which upregulates the osteogenesis inhibitor NF-κB and thus suppresses bone formation. Knockdown of METTL3 inhibited the degradation of IκBα and the S536 site phosphorylation of p65, thereby restraining NF-κB nuclear translocation and suppressing downstream transcription. More interestingly, ALKBH5 reversed these results by demethylase of MYD88-RNA (Yu et al., 2019a). A recent study showed that ALKBH5 affects osteogenesis by targeting BMP2 (Wang et al., 2020b) and TRAF4 (Cen et al., 2020). FTO also inhibits osteogenic differentiation of BMSCs through m6A demethylation (Zhang et al., 2020).

These studies indicate that METTL3-mediated m6A methylation could regulate bone formation at multiple levels and might provide new strategies for the treatment of osteoporosis. However, more studies are required to better understand the role of m6A methylation in regulating BMSCs and bone formation.

### 3.2 Regulating m6A Methylation on Osteoblasts

Studies have also shown that m6A methylation regulates osteoblast differentiation. Mi et al. (2020) discovered that downregulation of METTL3 promoted the osteogenic process *in vitro* and *in vivo* by inhibiting the maturation of miR-7212-5p. Further studies showed that miR-7212-5p inhibited osteoblast differentiation of MC3T3-E1 cells by targeting FGFR3. These findings suggest that METTL3 inhibits osteogenic‐related genes in MC3T3‐E1 cells. It seemed that METTL3 had a dual role in osteogenic differentiation, especially in different cell lines. FTO, an important RNA demethylase, also plays an important role in modulating osteoblast differentiation. Shen et al. (2018) found that FTO was upregulated during aging or osteoporosis in humans and mice, which upregulated BMSC differentiation into adipocytes and downregulated osteoblasts. Interestingly, conditional knockout of FTO in osteoblasts inhibited the progression of osteopenia in ovariectomy (OVX) mice but not in sham-operated mice. Mechanistically, GDF11 (growth differentiation factor 11)-FTO-PPARγ (peroxisome proliferator-activated receptor-gamma) signaling inhibits the differentiation of osteoblasts and promotes osteoporosis in humans and mice. Similarly, Zhang et al. (2019) found that conditional knockout of FTO in osteoblasts showed no difference in bone volume in 12-week-old mice compared to wild-type mice. However, 30-week-old mice with FTO knockout in osteoblasts had lower bone volume than wild-type mice. This phenomenon may be explained by the different animal models used. Additionally, Wang et al. (2019) studied the m6A methylome of the transcriptome in osteosarcoma cells by chemotherapy, indicating that m6A methylation modification may potentially affect the totipotency of osteosarcoma cells (OSCs) through the Wnt and Notch signaling pathways. Miao et al. (2019) also found that METTL3-mediated m6A methylation in OSCs promoted m6A levels of lymphoid enhancer factor-1 (LEF1) and upregulates the Wnt/β-catenin signaling pathway, which plays a critical role in osteoblast differentiation and osteogenesis (Wang et al., 2017; Zheng et al., 2020). These findings illustrated that m6A methylation affected osteoblast differentiation in humans and mice.

### 3.3 Regulating m6A Methylation on Osteoclasts

The bone resorption mediated by osteoclasts is important in bone metabolism. A recent study revealed that m6A methylation plays a prominent role in osteoclast differentiation and bone resorption (Salzman, 2016). The RNA methylase METTL3 affected m6A levels through the 1956 bp in circ_0008542 (noncoding RNA with a closed circular structure) and promoted the initiation of osteoclast-induced bone absorption. Circ_0008542 upregulated the competitive binding of miRNA-185-5p and promoted the expression of the target gene RANK. Instead, RNA demethylase ALKBH5 downregulated the combination of circ_0008542 with miRNA-185-5p to rescue excessive bone resorption (Wang et al., 2021). In addition, several studies have shown that m6A has a regulatory effect on intracellular inflammatory factors such as interleukin-1β (IL-1β), IL-6, interferon-gamma (IFN-γ), and tumor necrosis factor-α (TNF-α), leading to bone loss through the bone immune system (Neurath and Finotto, 2011; Briot and Roux, 2015; van Bodegraven and Bravenboer, 2019). Estrogen deficiency also increases inflammatory cytokines (Tsangari et al., 2004), followed by the activation of osteoclasts, increased bone resorption, and osteoporosis (Chen et al., 2017). Liu et al. (2019) also found that during the process of IL-1 β-induced chondrocyte inflammation, the expression level of METTL3 mRNA increased in a dose-dependent manner. At the same time, knockdown of METTL3 reduced the mRNA expression level of inflammatory factors in chondrocytes, including IL-6, IL-8, IL-12, and TNF-α, suggesting that m6A mRNA methylation promotes inflammatory injury in chondrocytes. Another study found that the knockdown of “reader” protein YTHDF2 increased the expression of MAP4K4 and MAP2K4, then activated MAPK and NF-κB signaling pathways, upregulated osteoclasts differentiation, and enhanced LPS-induced stimulation in RAW 264.7 cells (Yu et al., 2019b). These results suggest that m6A mRNA methylation plays a critical role in regulating osteoclasts through inflammatory responses.

## 4 Conclusion and Prospects

**FIGURE 1:**
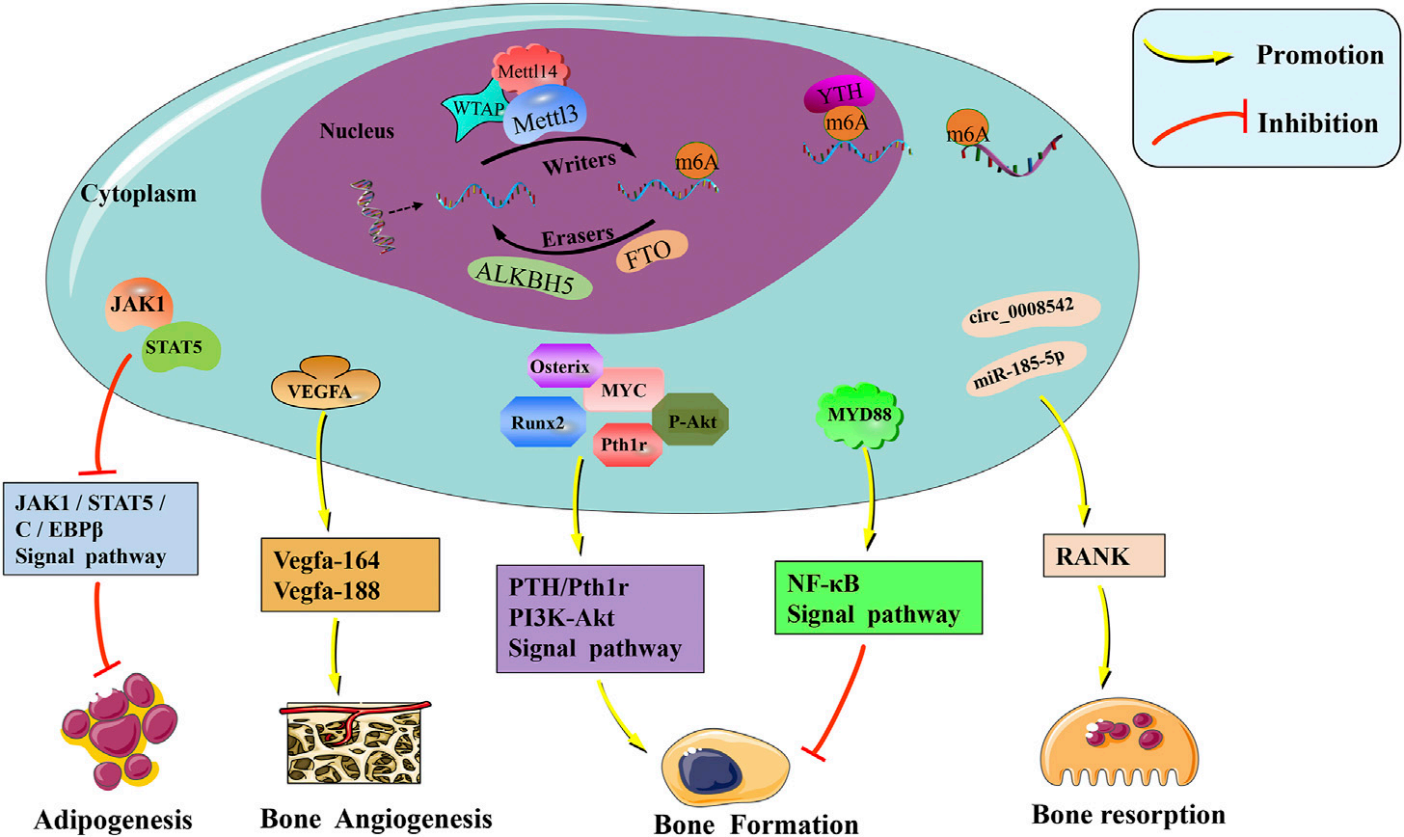
The molecular mechanism and physiological regulation roles of m6A modification in bone. M6A is mainly catalyzed by the METTL3–METTL14–WTAP methyltransferase complex, and the demethylases ALKBH5 and FTO remove the methyl group of m6A off RNAs. Readers of the YTH domain family are effectors that recognize the m6A methylation code and convert it into signals. M6A modification regulates the expression of Runx2, Osterix, VEGF, RANK, and other related genes affecting bone metabolism. Furthermore, PTH/Pth1r, PI3K-Akt, NF-κB, and other signaling pathways were also mediated by m6A, which is important in the regulation of bone homeostasis.

In summary, m6A methylation regulated osteogenic differentiation and bone metabolism. But the function of m6A methylation maybe like a “double-edged sword,” by which it can either promote or inhibit bone formation in different ways (Figure 1; Table 1). Undoubtedly, m6A regulation has provided novel insight into the molecular mechanism of bone metabolism.

**TABLE 1:** Multiple functions exerted by m6A RNA methylation in bone.

m6A component	m6A levels	Related targets	Biological function	Sample resources	Refs
Mettl3 knockout	Low	Pth1r ↓	Inhibit osteogenesis	BMSCs	Wu et al. (2018)
Mettl3 knockdown	Low	JAK1 ↑	Inhibit osteogenesis	BMSCs	Yao et al. (2019)
Mettl3 knockdown	Low	Vegfa-164 ↓	Inhibit osteogenesis	BMSCs	Tian et al. (2019)
		Vegfa-188 ↓			
Mettl3 knockdown	Low	MYD88 ↓	Promote osteogenesis	MenSCs	Yu et al. (2019)
Mettl3 knockdown	Low	miR-320 ↑	Inhibit osteogenesis	BMSCs	Yan et al. (2019)
		RUNX2 ↓			
Mettl3 knockdown	Low	miR-7212-5p ↓	Promote osteogenesis	MC3T3	Mi et al. (2020)
Mettl3 knockdown or ALKBH5 overexpression	Low	circ_0008542 ↓	Inhibit bone resorption	Osteoclast	Wang et al. (2021)
		RANK↓			
YTHDF2 knockdown	—	MAP2K4 ↑	—	Raw264.7	Yu et al. (2019)
		MAP4K4 ↑			
ALKBH5 knockdown	high	TRAF4 ↓	Inhibit osteogenesis	MSC	Cen et al. (2020)
ALKBH5 knockdown	high	BMP2 ↓	Inhibit osteogenesis	OLF	Wang et al. (2020)
		P-AKT ↓			
FTO knockdown	high	PPARγ ↓	Promote osteogenesis	BMSCs	Shen et al. (2018)
FTO knockdown	high	MYC ↑	Promote osteogenesis	BMSCs	Zhang et al. (2020)
FTO knockout	high	Hspa1a ↓	Inhibit osteogenesis	Osteoblast	Zhang et al. (2019)

However, the study of m6A modification on bone metabolism is still in its infancy. First, existing research on m6A in bone mainly focused on Writers; the mechanism of m6A Erasers and Readers in bone metabolism require further study. The methylation of m6A is a dynamic and reversible process, and how the Writers and Erasers coordinate and how the Readers play their role after recognizing RNA methylation needs further exploration. Second, osteoclast-mediated bone resorption is also an important part of bone metabolism, but there are few related studies. Moreover, although METTL3 targets Runx2, VEGF and different signaling pathways to promote osteogenic differentiation, it remains controversial whether METTL3 is a potential therapeutic target for osteoporosis, as METTL3 also activates osteoclasts and then increases bone resorption. Due to the complexity of regulating m6A methylation in bone metabolism, further studies are needed to explore its underlying mechanism.

## References

[B1] Akhavan-Niaki H. Samadani A. A. (2013). DNA Methylation and Cancer Development: Molecular Mechanism. Cell Biochem Biophys 67 (2), 501–513. 10.1007/s12013-013-9555-2 23508887

[B2] Alarcón C. R. Goodarzi H. Lee H. Liu X. Tavazoie S. Tavazoie S. F. (2015). HNRNPA2B1 Is a Mediator of m6A-dependent Nuclear RNA Processing Events. Cell 162 (6), 1299–1308. 10.1016/j.cell.2015.08.011 26321680 PMC4673968

[B3] Bliuc D. Alarkawi D. Nguyen T. V. Eisman J. A. Center J. R. (2015). Risk of Subsequent Fractures and Mortality in Elderly Women and Men with Fragility Fractures with and without Osteoporotic Bone Density: the Dubbo Osteoporosis Epidemiology Study. J. Bone Miner Res. 30 (4), 637–646. 10.1002/jbmr.2393 25359586

[B4] Breier G. Albrecht U. Sterrer S. Risau W. (1992). Expression of Vascular Endothelial Growth Factor during Embryonic Angiogenesis and Endothelial Cell Differentiation. Development 114 (2), 521–532. 10.1242/dev.114.2.521 1592003

[B5] Briot K. Roux C. (2015). Inflammation, Bone Loss and Fracture Risk in Spondyloarthritis: Figure 1. RMD Open 1 (1), e000052. 10.1136/rmdopen-2015-000052 26509065 PMC4613172

[B6] Cai X. Wang X. Cao C. Gao Y. Zhang S. Yang Z. (2018). HBXIP-elevated Methyltransferase METTL3 Promotes the Progression of Breast Cancer via Inhibiting Tumor Suppressor Let-7g. Cancer Lett. 415, 11–19. 10.1016/j.canlet.2017.11.018 29174803

[B7] Carmeliet P. Ng Y.-S. Nuyens D. Theilmeier G. Brusselmans K. Cornelissen I. (1999). Impaired Myocardial Angiogenesis and Ischemic Cardiomyopathy in Mice Lacking the Vascular Endothelial Growth Factor Isoforms VEGF164 and VEGF188. Nat. Med. 5 (5), 495–502. 10.1038/8379 10229225

[B8] Cen S. Li J. Cai Z. Pan Y. Sun Z. Li Z. (2020). TRAF4 Acts as a Fate Checkpoint to Regulate the Adipogenic Differentiation of MSCs by Activating PKM2. EBioMedicine 54, 102722. 10.1016/j.ebiom.2020.102722 32268273 PMC7191261

[B9] Chen Q. Shou P. Zheng C. Jiang M. Cao G. Yang Q. (2016). Fate Decision of Mesenchymal Stem Cells: Adipocytes or Osteoblasts? Cell Death Differ 23 (7), 1128–1139. 10.1038/cdd.2015.168 26868907 PMC4946886

[B10] Chen X. Zhi X. Pan P. Cui J. Cao L. Weng W. (2017). Matrine Prevents Bone Loss in Ovariectomized Mice by Inhibiting RANKL‐induced Osteoclastogenesis. FASEB j. 31 (11), 4855–4865. 10.1096/fj.201700316R 28739641 PMC5636701

[B11] Chen Y. Peng C. Chen J. Chen D. Yang B. He B. (2019). WTAP Facilitates Progression of Hepatocellular Carcinoma via m6A-HuR-dependent Epigenetic Silencing of ETS1. Mol. Cancer 18 (1), 127. 10.1186/s12943-019-1053-8 31438961 PMC6704583

[B12] Desrosiers R. Friderici K. Rottman F. (1974). Identification of Methylated Nucleosides in Messenger RNA from Novikoff Hepatoma Cells. Proc. Natl. Acad. Sci. 71 (10), 3971–3975. 10.1073/pnas.71.10.3971 4372599 PMC434308

[B13] Dina C. Meyre D. Gallina S. Durand E. Körner A. Jacobson P. (2007). Variation in FTO Contributes to Childhood Obesity and Severe Adult Obesity. Nat. Genet. 39 (6), 724–726. 10.1038/ng2048 17496892

[B14] Dominissini D. Moshitch-Moshkovitz S. Schwartz S. Salmon-Divon M. Ungar L. Osenberg S. (2012). Topology of the Human and Mouse m6A RNA Methylomes Revealed by m6A-Seq. Nature 485 (7397), 201–206. 10.1038/nature11112 22575960

[B15] Du M. Zhang Y. Mao Y. Mou J. Zhao J. Xue Q. (2017). MiR-33a Suppresses Proliferation of NSCLC Cells via Targeting METTL3 mRNA. Biochem. Biophysical Res. Commun. 482 (4), 582–589. 10.1016/j.bbrc.2016.11.077 27856248

[B16] Felsenberg D. Boonen S. (2005). The Bone Quality Framework: Determinants of Bone Strength and Their Interrelationships, and Implications for Osteoporosis Management. Clin. Ther. 27 (1), 1–11. 10.1016/j.clinthera.2004.12.020 15763602

[B17] Feng Z. Li Q. Meng R. Yi B. Xu Q. (2018). METTL 3 Regulates Alternative Splicing of MyD88 upon the Lipopolysaccharide‐induced Inflammatory Response in Human Dental Pulp Cells. J. Cel Mol Med 22 (5), 2558–2568. 10.1111/jcmm.13491 PMC5908103 29502358

[B18] Guo M. Liu X. Zheng X. Huang Y. Chen X. (2017). m6A RNA Modification Determines Cell Fate by Regulating mRNA DegradationA RNA Modification Determines Cell Fate by Regulating mRNA Degradation. Cell Reprogramming 19 (4), 225–231. 10.1089/cell.2016.0041 28682669

[B19] Haussmann I. U. Bodi Z. Sanchez-Moran E. Mongan N. P. Archer N. Fray R. G. (2016). m6A Potentiates Sxl Alternative Pre-mRNA Splicing for Robust Drosophila Sex determinationA Potentiates Sxl Alternative Pre-mRNA Splicing for Robust Drosophila Sex Determination. Nature 540 (7632), 301–304. 10.1038/nature20577 27919081

[B20] Helm M. Motorin Y. (2017). Detecting RNA Modifications in the Epitranscriptome: Predict and Validate. Nat. Rev. Genet. 18 (5), 275–291. 10.1038/nrg.2016.169 28216634

[B21] Hu K. Olsen B. R. (2016). Osteoblast-derived VEGF Regulates Osteoblast Differentiation and Bone Formation during Bone Repair. J. Clin. Invest. 126 (2), 509–526. 10.1172/JCI82585 26731472 PMC4731163

[B22] Huang H. Weng H. Chen J. (2020). m6A Modification in Coding and Non-coding RNAs: Roles and Therapeutic Implications in CancerA Modification in Coding and Non-coding RNAs: Roles and Therapeutic Implications in Cancer. Cancer Cell 37 (3), 270–288. 10.1016/j.ccell.2020.02.004 32183948 PMC7141420

[B23] Huang H. Weng H. Sun W. Qin X. Shi H. Wu H. (2018). Recognition of RNA N6-Methyladenosine by IGF2BP Proteins Enhances mRNA Stability and Translation. Nat. Cel Biol 20 (3), 285–295. 10.1038/s41556-018-0045-z PMC5826585 29476152

[B24] Jia G. Fu Y. Zhao X. Dai Q. Zheng G. Yang Y. (2011). N6-methyladenosine in Nuclear RNA Is a Major Substrate of the Obesity-Associated FTO. Nat. Chem. Biol. 7 (12), 885–887. 10.1038/nchembio.687 22002720 PMC3218240

[B25] Kasowitz S. D. Ma J. Anderson S. J. Leu N. A. Xu Y. Gregory B. D. (2018). Nuclear m6A Reader YTHDC1 Regulates Alternative Polyadenylation and Splicing during Mouse Oocyte Development. Plos Genet. 14 (5), e1007412. 10.1371/journal.pgen.1007412 29799838 PMC5991768

[B26] Kawai M. Devlin M. J. Rosen C. J. (2009). Fat Targets for Skeletal Health. Nat. Rev. Rheumatol. 5 (7), 365–372. 10.1038/nrrheum.2009.102 19468288 PMC3661210

[B27] Ke S. Pandya-Jones A. Saito Y. Fak J. J. Vågbø C. B. Geula S. (2017). m6A mRNA Modifications Are Deposited in Nascent Pre-mRNA and Are Not Required for Splicing but Do Specify Cytoplasmic turnoverA mRNA Modifications Are Deposited in Nascent Pre-mRNA and Are Not Required for Splicing but Do Specify Cytoplasmic Turnover. Genes Dev. 31 (10), 990–1006. 10.1101/gad.301036.117 28637692 PMC5495127

[B28] Knuckles P. Lence T. Haussmann I. U. Jacob D. Kreim N. Carl S. H. (2018). Zc3h13/Flacc Is Required for Adenosine Methylation by Bridging the mRNA-Binding Factor Rbm15/Spenito to the m6A Machinery Component Wtap/Fl(2)d. Genes Dev. 32 (5-6), 415–429. 10.1101/gad.309146.117 29535189 PMC5900714

[B29] Kohli R. M. Zhang Y. (2013). TET Enzymes, TDG and the Dynamics of DNA Demethylation. Nature 502 (7472), 472–479. 10.1038/nature12750 24153300 PMC4046508

[B30] Lin S. Liu J. Jiang W. Wang P. Sun C. Wang X. (2019). METTL3 Promotes the Proliferation and Mobility of Gastric Cancer Cells. Open Med. (Wars) 14, 25–31. 10.1515/med-2019-0005 30886897 PMC6419388

[B31] Litt M. D. Simpson M. Gaszner M. Allis C. D. Felsenfeld G. (2001). Correlation between Histone Lysine Methylation and Developmental Changes at the Chicken β-Globin Locus. Science 293 (5539), 2453–2455. 10.1126/science.1064413 11498546

[B32] Liu N. Dai Q. Zheng G. He C. Parisien M. Pan T. (2015). N6-methyladenosine-dependent RNA Structural Switches Regulate RNA-Protein Interactions. Nature 518 (7540), 560–564. 10.1038/nature14234 25719671 PMC4355918

[B33] Liu Q. Li M. Jiang L. Jiang R. Fu B. (2019). METTL3 Promotes Experimental Osteoarthritis Development by Regulating Inflammatory Response and Apoptosis in Chondrocyte. Biochem. Biophysical Res. Commun. 516 (1), 22–27. 10.1016/j.bbrc.2019.05.168 31186141

[B34] Luo S. Tong L. (2014). Molecular Basis for the Recognition of Methylated Adenines in RNA by the Eukaryotic YTH Domain. Proc. Natl. Acad. Sci. 111 (38), 13834–13839. 10.1073/pnas.1412742111 25201973 PMC4183320

[B35] Marie P. J. (2012). Signaling Pathways Affecting Skeletal Health. Curr. Osteoporos. Rep. 10 (3), 190–198. 10.1007/s11914-012-0109-0 22711369

[B36] Meyer K. D. Saletore Y. Zumbo P. Elemento O. Mason C. E. Jaffrey S. R. (2012). Comprehensive Analysis of mRNA Methylation Reveals Enrichment in 3′ UTRs and Near Stop Codons. Cell 149 (7), 1635–1646. 10.1016/j.cell.2012.05.003 22608085 PMC3383396

[B37] Mi B. Xiong Y. Yan C. Chen L. Xue H. Panayi A. C. (2020). Methyltransferase‐like 3‐mediated N6‐methyladenosine Modification of miR‐7212‐5p Drives Osteoblast Differentiation and Fracture Healing. J. Cel Mol Med 24 (11), 6385–6396. 10.1111/jcmm.15284 PMC7294157 32307908

[B38] Miao W. Chen J. Jia L. Ma J. Song D. (2019). The m6A Methyltransferase METTL3 Promotes Osteosarcoma Progression by Regulating the m6A Level of LEF1. Biochem. Biophysical Res. Commun. 516 (3), 719–725. 10.1016/j.bbrc.2019.06.128 31253399

[B39] Muftic M. Selimovic E. Miladinovic K. (2013). Osteoporosis - Comparative Study between Quantitative Ultrasound of Calcaneus and DXA. Med. Arh 67 (4), 289–291. 10.5455/medarh.2013.67.289-291 24520757

[B40] Neurath M. F. Finotto S. (2011). IL-6 Signaling in Autoimmunity, Chronic Inflammation and Inflammation-Associated Cancer. Cytokine Growth Factor. Rev. 22 (2), 83–89. 10.1016/j.cytogfr.2011.02.003 21377916

[B41] NIH Consensus Development Panel on Osteoporosis Prevention, Diagnosis, and Therapy, March 7-29, 2000: Highlights of the Conference. South. Med. J. (2001). 94(6):569–573. 10.1001/jama.285.6.785 11440324

[B42] Panneerdoss S. Eedunuri V. K. Yadav P. Timilsina S. Rajamanickam S. Viswanadhapalli S. (2018). Cross-talk Among Writers, Readers, and Erasers of M 6 A Regulates Cancer Growth and Progression. Sci. Adv. 4 (10), eaar8263. 10.1126/sciadv.aar8263 30306128 PMC6170038

[B43] Patil D. P. Chen C.-K. Pickering B. F. Chow A. Jackson C. Guttman M. (2016). m6A RNA Methylation Promotes XIST-Mediated Transcriptional repressionA RNA Methylation Promotes XIST-Mediated Transcriptional Repression. Nature 537 (7620), 369–373. 10.1038/nature19342 27602518 PMC5509218

[B44] Ping X.-L. Sun B.-F. Wang L. Xiao W. Yang X. Wang W.-J. (2014). Mammalian WTAP Is a Regulatory Subunit of the RNA N6-Methyladenosine Methyltransferase. Cell Res 24 (2), 177–189. 10.1038/cr.2014.3 24407421 PMC3915904

[B45] Qing Y. Dong L. Gao L. Li C. Li Y. Han L. (2021). R-2-hydroxyglutarate Attenuates Aerobic Glycolysis in Leukemia by Targeting the FTO/m6A/PFKP/LDHB axis. Mol. Cel 81 (5), 922–939. 10.1016/j.molcel.2020.12.026 PMC7935770 33434505

[B46] Ramasamy S. K. Kusumbe A. P. Wang L. Adams R. H. (2014). Endothelial Notch Activity Promotes Angiogenesis and Osteogenesis in Bone. Nature 507 (7492), 376–380. 10.1038/nature13146 24647000 PMC4943529

[B47] Roignant J.-Y. Soller M. (2017). m 6 A in mRNA: An Ancient Mechanism for Fine-Tuning Gene ExpressionA in mRNA: An Ancient Mechanism for Fine-Tuning Gene Expression. Trends Genet. 33 (6), 380–390. 10.1016/j.tig.2017.04.003 28499622

[B48] Rosen C. J. Ackert-Bicknell C. Rodriguez J. P. Pino A. M. (2009). Marrow Fat and the Bone Microenvironment: Developmental, Functional, and Pathological Implications. Crit. Rev. Eukar Gene Expr. 19 (2), 109–124. 10.1615/critreveukargeneexpr.v19.i2.20 PMC2674609 19392647

[B49] Salzman J. (2016). Circular RNA Expression: Its Potential Regulation and Function. Trends Genet. 32 (5), 309–316. 10.1016/j.tig.2016.03.002 27050930 PMC4948998

[B50] Scheller E. L. Rosen C. J. (2014). What's the Matter with MAT? Marrow Adipose Tissue, Metabolism, and Skeletal Health. Ann. N.Y. Acad. Sci. 1311, 14–30. 10.1111/nyas.12327 24650218 PMC4049420

[B51] Schwartz S. Mumbach M. R. Jovanovic M. Wang T. Maciag K. Bushkin G. G. (2014). Perturbation of m6A Writers Reveals Two Distinct Classes of mRNA Methylation at Internal and 5′ Sites. Cel Rep. 8 (1), 284–296. 10.1016/j.celrep.2014.05.048 PMC4142486 24981863

[B52] Shapiro C. L. Van Poznak C. Lacchetti C. Kirshner J. Eastell R. Gagel R. (2019). Management of Osteoporosis in Survivors of Adult Cancers with Nonmetastatic Disease: ASCO Clinical Practice Guideline. Jco 37, 2916–2946. 10.1200/JCO.19.01696 31532726

[B53] Shen G.-S. Zhou H.-B. Zhang H. Chen B. Liu Z.-P. Yuan Y. (2018). The GDF11-FTO-Pparγ axis Controls the Shift of Osteoporotic MSC Fate to Adipocyte and Inhibits Bone Formation during Osteoporosis. Biochim. Biophys. Acta (Bba) - Mol. Basis Dis. 1864 (12), 3644–3654. 10.1016/j.bbadis.2018.09.015 30279140

[B54] Shi H. Wang X. Lu Z. Zhao B. S. Ma H. Hsu P. J. (2017). YTHDF3 Facilitates Translation and Decay of N6-Methyladenosine-Modified RNA. Cel Res 27 (3), 315–328. 10.1038/cr.2017.15 PMC5339834 28106072

[B55] Shi H. Wei J. He C. (2019). Where, when, and How: Context-dependent Functions of RNA Methylation Writers, Readers, and Erasers. Mol. Cel 74 (4), 640–650. 10.1016/j.molcel.2019.04.025 PMC6527355 31100245

[B56] Tian C. Huang Y. Li Q. Feng Z. Xu Q. (2019). Mettl3 Regulates Osteogenic Differentiation and Alternative Splicing of Vegfa in Bone Marrow Mesenchymal Stem Cells. Ijms 20 (3), 551. 10.3390/ijms20030551 PMC6387109 30696066

[B57] Tian L. Yang R. Wei L. Liu J. Yang Y. Shao F. (2017). Prevalence of Osteoporosis and Related Lifestyle and Metabolic Factors of Postmenopausal Women and Elderly Men. Medicine (Baltimore) 96 (43), e8294. 10.1097/MD.0000000000008294 29068999 PMC5671832

[B58] Tong X. Chen X. Zhang S. Huang M. Shen X. Xu J. (2019). The Effect of Exercise on the Prevention of Osteoporosis and Bone Angiogenesis. Biomed. Res. Int. 2019, 1–8. 10.1155/2019/8171897 PMC6500645 31139653

[B59] Tsangari H. Findlay D. M. Kuliwaba J. S. Atkins G. J. Fazzalari N. L. (2004). Increased Expression of IL-6 and RANK mRNA in Human Trabecular Bone from Fragility Fracture of the Femoral Neck. Bone 35 (1), 334–342. 10.1016/j.bone.2004.02.006 15207775

[B60] van Bodegraven A. A. Bravenboer N. (2019). Perspective on Skeletal Health in Inflammatory Bowel Disease. Osteoporos. Int. 31, 637–646. 10.1007/s00198-019-05234-w 31822927 PMC7075921

[B61] Wallner C. Schira J. Wagner J. M. Schulte M. Fischer S. Hirsch T. (2015). Application of VEGFA and FGF-9 Enhances Angiogenesis, Osteogenesis and Bone Remodeling in Type 2 Diabetic Long Bone Regeneration. PLoS One 10 (3), e0118823. 10.1371/journal.pone.0118823 25742620 PMC4350939

[B62] Wang H.-F. Kuang M.-j. Han S.-j. Wang A.-b. Qiu J. Wang F. (2020). BMP2 Modified by the m6A Demethylation Enzyme ALKBH5 in the Ossification of the Ligamentum Flavum through the AKT Signaling Pathway. Calcif Tissue Int. 106 (5), 486–493. 10.1007/s00223-019-00654-6 31897529

[B63] Wang L. Song C. Wang N. Li S. Liu Q. Sun Z. (2020). NADP Modulates RNA m6A Methylation and Adipogenesis via Enhancing FTO Activity. Nat. Chem. Biol. 16 (12), 1394–1402. 10.1038/s41589-020-0601-2 32719557

[B64] Wang W. Qiao S.-C. Wu X.-B. Sun B. Yang J.-G. Li X. (2021). Circ_0008542 in Osteoblast Exosomes Promotes Osteoclast-Induced Bone Resorption through m6A Methylation. Cell Death Dis 12 (7), 628. 10.1038/s41419-021-03915-1 34145224 PMC8213782

[B65] Wang X. Feng J. Xue Y. Guan Z. Zhang D. Liu Z. (2016). Structural Basis of N6-Adenosine Methylation by the METTL3-METTL14 Complex. Nature 534 (7608), 575–578. 10.1038/nature18298 27281194

[B66] Wang X. Lu Z. Gomez A. Hon G. C. Yue Y. Han D. (2014). N6-methyladenosine-dependent Regulation of Messenger RNA Stability. Nature 505 (7481), 117–120. 10.1038/nature12730 24284625 PMC3877715

[B67] Wang X. Zhao B. S. Roundtree I. A. Lu Z. Han D. Ma H. (2015). N6-methyladenosine Modulates Messenger RNA Translation Efficiency. Cell 161 (6), 1388–1399. 10.1016/j.cell.2015.05.014 26046440 PMC4825696

[B68] Wang Y. Zeng L. Liang C. Zan R. Ji W. Zhang Z. (2019). Integrated Analysis of Transcriptome-wide m6A Methylome of Osteosarcoma Stem Cells Enriched by Chemotherapy. Epigenomics 11 (15), 1693–1715. 10.2217/epi-2019-0262 31650864

[B69] Wang Y. Zhang X. Shao J. Liu H. Liu X. Luo E. (2017). Adiponectin Regulates BMSC Osteogenic Differentiation and Osteogenesis through the Wnt/β-Catenin Pathway. Sci. Rep. 7 (1), 3652. 10.1038/s41598-017-03899-z 28623357 PMC5473871

[B70] Warda A. S. Kretschmer J. Hackert P. Lenz C. Urlaub H. Höbartner C. (2017). Human METTL16 Is a N 6 ‐methyladenosine (M 6 A) Methyltransferase that Targets pre‐mRNAs and Various Non‐coding RNAs. EMBO Rep. 18 (11), 2004–2014. 10.15252/embr.201744940 29051200 PMC5666602

[B71] Wei C.-M. Gershowitz A. Moss B. (1975). Methylated Nucleotides Block 5′ Terminus of HeLa Cell Messenger RNA. Cell 4 (4), 379–386. 10.1016/0092-8674(75)90158-0 164293

[B72] Wen J. Lv R. Ma H. Shen H. He C. Wang J. (2018). Zc3h13 Regulates Nuclear RNA m6A Methylation and Mouse Embryonic Stem Cell Self-Renewal. Mol. Cel 69 (6), 1028–1038. 10.1016/j.molcel.2018.02.015 PMC5858226 29547716

[B73] Wu Y. Xie L. Wang M. Xiong Q. Guo Y. Liang Y. (2018). Mettl3-mediated m6A RNA Methylation Regulates the Fate of Bone Marrow Mesenchymal Stem Cells and Osteoporosis. Nat. Commun. 9 (1), 4772. 10.1038/s41467-018-06898-4 30429466 PMC6235890

[B74] Xu C. Wang X. Liu K. Roundtree I. A. Tempel W. Li Y. (2014). Structural Basis for Selective Binding of m6A RNA by the YTHDC1 YTH Domain. Nat. Chem. Biol. 10 (11), 927–929. 10.1038/nchembio.1654 25242552

[B75] Xu H. Yi Q. Yang C. Wang Y. Tian J. Zhu J. (2016). Histone Modifications Interact with DNA Methylation at the GATA4 Promoter during Differentiation of Mesenchymal Stem Cells into Cardiomyocyte-like Cells. Cell Prolif. 49 (3), 315–329. 10.1111/cpr.12253 27117983 PMC6496691

[B76] Xu K. Yang Y. Feng G.-H. Sun B.-F. Chen J.-Q. Li Y.-F. (2017). Mettl3-mediated m6A Regulates Spermatogonial Differentiation and Meiosis Initiation. Cel Res 27 (9), 1100–1114. 10.1038/cr.2017.100 PMC5587845 28809392

[B77] Yan G. Yuan Y. He M. Gong R. Lei H. Zhou H. (2020). m6A Methylation of Precursor-miR-320/runx2 Controls Osteogenic Potential of Bone Marrow-Derived Mesenchymal Stem CellsA Methylation of Precursor-miR-320/runx2 Controls Osteogenic Potential of Bone Marrow-Derived Mesenchymal Stem Cells. Mol. Ther. - Nucleic Acids 19, 421–436. 10.1016/j.omtn.2019.12.001 31896070 PMC6940653

[B78] Yao Y. Bi Z. Wu R. Zhao Y. Liu Y. Liu Q. (2019). METTL3 Inhibits BMSC Adipogenic Differentiation by Targeting the JAK1/STAT5/C/EBPβ Pathway via an M 6 A‐YTHDF2-dependent Manner. FASEB j. 33 (6), 7529–7544. 10.1096/fj.201802644R 30865855

[B79] Yu J. Chen M. Huang H. Zhu J. Song H. Zhu J. (2018). Dynamic m6A Modification Regulates Local Translation of mRNA in Axons. Nucleic Acids Res. 46 (3), 1412–1423. 10.1093/nar/gkx1182 29186567 PMC5815124

[B80] Yu J. Shen L. Liu Y. Ming H. Zhu X. Chu M. (2019). The m6A Methyltransferase METTL3 Cooperates with Demethylase ALKBH5 to Regulate Osteogenic Differentiation through NF-Κb Signaling. Mol. Cel Biochem 463, 203–210. 10.1007/s11010-019-03641-5 31643040

[B81] Yu R. Li Q. Feng Z. Cai L. Xu Q. (2019). m6A Reader YTHDF2 Regulates LPS-Induced Inflammatory Response. Ijms 20 (6), 1323. 10.3390/ijms20061323 PMC6470741 30875984

[B82] Zhang C. Samanta D. Lu H. Bullen J. W. Zhang H. Chen I. (2016). Hypoxia Induces the Breast Cancer Stem Cell Phenotype by HIF-dependent and ALKBH5-Mediated m6A-Demethylation of NANOG mRNA. Proc. Natl. Acad. Sci. USA 113 (14), E2047–E2056. 10.1073/pnas.1602883113 27001847 PMC4833258

[B83] Zhang F. Kang Y. Wang M. Li Y. Xu T. Yang W. (2018). Fragile X Mental Retardation Protein Modulates the Stability of its m6A-Marked Messenger RNA Targets. Hum. Mol. Genet. 27 (22), 3936–3950. 10.1093/hmg/ddy292 30107516 PMC6216232

[B84] Zhang Q. Riddle R. C. Yang Q. Rosen C. R. Guttridge D. C. Dirckx N. (2019). The RNA Demethylase FTO Is Required for Maintenance of Bone Mass and Functions to Protect Osteoblasts from Genotoxic Damage. Proc. Natl. Acad. Sci. USA 116 (36), 17980–17989. 10.1073/pnas.1905489116 31434789 PMC6731662

[B85] Zhang X. Wang Y. Zhao H. Han X. Zhao T. Qu P. (2020). Extracellular Vesicle-Encapsulated miR-22-3p from Bone Marrow Mesenchymal Stem Cell Promotes Osteogenic Differentiation via FTO Inhibition. Stem Cel Res Ther 11 (1), 227. 10.1186/s13287-020-01707-6 PMC7285613 32522250

[B86] Zhao X. Yang Y. Sun B.-F. Zhao Y.-L. Yang Y.-G. (2014). FTO and Obesity: Mechanisms of Association. Curr. Diab Rep. 14 (5), 486. 10.1007/s11892-014-0486-0 24627050

[B87] Zheng G. Dahl J. A. Niu Y. Fedorcsak P. Huang C.-M. Li C. J. (2013). ALKBH5 Is a Mammalian RNA Demethylase that Impacts RNA Metabolism and Mouse Fertility. Mol. Cel 49 (1), 18–29. 10.1016/j.molcel.2012.10.015 PMC3646334 23177736

[B88] Zheng W. Ding B. Li X. Liu D. Yokota H. Zhang P. (2020). Knee Loading Repairs Osteoporotic Osteoarthritis by Relieving Abnormal Remodeling of Subchondral Bone via Wnt/β‐catenin Signaling. FASEB j. 34, 3399–3412. 10.1096/fj.201902117R 31925860 PMC7018573

